# Poly[1*H*-imidazol-3-ium [di-μ-nitrato-sodium]]

**DOI:** 10.1107/S1600536813011951

**Published:** 2013-05-11

**Authors:** Chahrazed Trifa, Amira Bouhali, Sofiane Bouacida, Chaouki Boudaren, Thierry Bataille

**Affiliations:** a Unité de Recherche de Chimie de l’Environnement et Moléculaire Structurale, CHEMS, Université Constantine, 25000, Algeria; b Département Sciences de la Matière, Faculté des Sciences Exactes et Sciences de la Nature et de la Vie, Université Oum El Bouaghi, 04000 Oum El Bouaghi, Algeria; c Sciences Chimiques de Rennes, UMR 6226 CNRS - Université de Rennes 1, Avenue du Général Leclerc, 35042 Rennes Cedex, France

## Abstract

In the title compound {(C_3_H_5_N_2_)[Na(NO_3_)_2_]}_*n*_, the Na^I^ ion is coordinated by eight O atoms from three bidentate nitrate anions and two O atoms from two monodentate nitrate anions, displaying a bicapped trigonal–prismatic geometry. The imidazolium cation is essentially planar (r.m.s. deviation for all non-H atoms = 0.0018 Å). In the crystal, the Na^I^ ions are connected by bridging nitrate ligands, forming layers parallel to (010). The imidazolium cations are sandwiched between these layers. Weak C—H⋯O hydrogen bonds link the layers into a three-dimensional network. In addtion, π–π inter­actions between the imidazolium rings [centroid–centroid distance = 3.588 (3) Å] are observed.

## Related literature

For applications of imidazole and its derivatives, see: Huang *et al.* (2008 ▶, 2011 ▶). For the preparation and characterization of some metal complexes of imidazolium, see: Gao *et al.* (2009 ▶); Zhang *et al.* (2011 ▶); Zhu (2012 ▶); Han *et al.* (2007 ▶); Wenyan *et al.* (2011 ▶). 
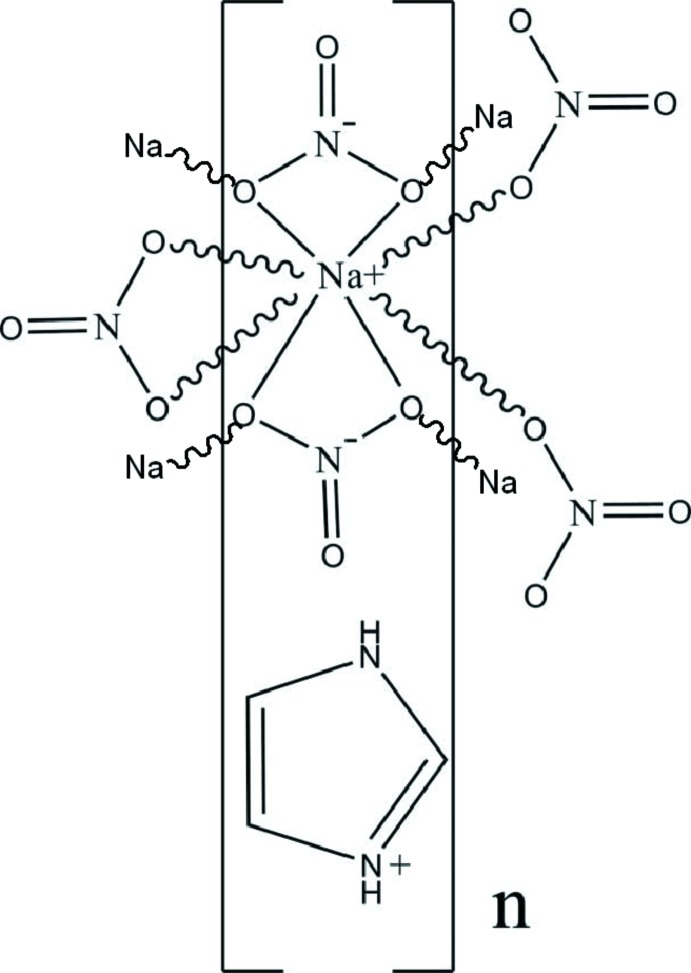


## Experimental

### 

#### Crystal data

(C_3_H_5_N_2_)[Na(NO_3_)_2_]
*M* *_r_* = 216.1
Monoclinic, 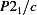

*a* = 3.5875 (3) Å
*b* = 24.8548 (17) Å
*c* = 8.819 (6) Å
β = 95.546 (4)°
*V* = 782.7 (5) Å^3^
*Z* = 4
Mo *K*α radiation
μ = 0.22 mm^−1^
*T* = 150 K
0.52 × 0.15 × 0.13 mm

#### Data collection

Nonius KappaCCD diffractometer
Absorption correction: multi-scan (*SADABS*; Sheldrick, 2002 ▶) *T*_min_ = 0.884, *T*_max_ = 0.972
9892 measured reflections
1778 independent reflections
1541 reflections with *I* > 2σ(*I*)
*R*_int_ = 0.029

#### Refinement

*R*[*F* ^2^ > 2σ(*F* ^2^)] = 0.034
*wR*(*F* ^2^) = 0.084
*S* = 1.10
1778 reflections
127 parameters
H-atom parameters constrained
Δρ_max_ = 0.19 e Å^−3^
Δρ_min_ = −0.29 e Å^−3^

### 

Data collection: *COLLECT* (Nonius, 1998 ▶); cell refinement: *SCALEPACK* (Otwinowski & Minor, 1997 ▶); data reduction: *DENZO* (Otwinowski & Minor, 1997 ▶) and *SCALEPACK*; program(s) used to solve structure: *SIR2002* (Burla *et al.*, 2003 ▶); program(s) used to refine structure: *SHELXL97* (Sheldrick, 2008 ▶); molecular graphics: *ORTEP-3 for Windows* (Farrugia, 2012 ▶) and *DIAMOND* (Brandenburg & Berndt, 2001 ▶); software used to prepare material for publication: *WinGX* (Farrugia, 2012 ▶) and *CRYSCAL* (T. Roisnel, local program).

## Supplementary Material

Crystal structure: contains datablock(s) global, I. DOI: 10.1107/S1600536813011951/lh5609sup1.cif

Structure factors: contains datablock(s) I. DOI: 10.1107/S1600536813011951/lh5609Isup2.hkl

Additional supplementary materials:  crystallographic information; 3D view; checkCIF report

## Figures and Tables

**Table 1:** Selected bond lengths (Å)

Na1—O12	2.4321 (16)
Na1—O21	2.4639 (14)
Na1—O13^i^	2.5106 (13)
Na1—O23	2.5338 (13)
Na1—O13^ii^	2.5730 (14)
Na1—O11^ii^	2.5910 (19)
Na1—O11	2.6239 (17)
Na1—O21^iii^	2.6776 (14)

Symmetry codes: (i) 
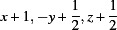
; (ii) 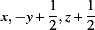; (iii) 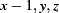.

**Table 2:** Hydrogen-bond geometry (Å, °)

*D*—H⋯*A*	*D*—H	H⋯*A*	*D*⋯*A*	*D*—H⋯*A*
C1—H1⋯O23^iv^	0.95	2.50	3.438 (3)	168
C4—H4⋯O11^v^	0.95	2.41	3.355 (3)	173

Symmetry codes: (iv) 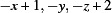; (v) 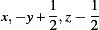.

**Fig. 1.**
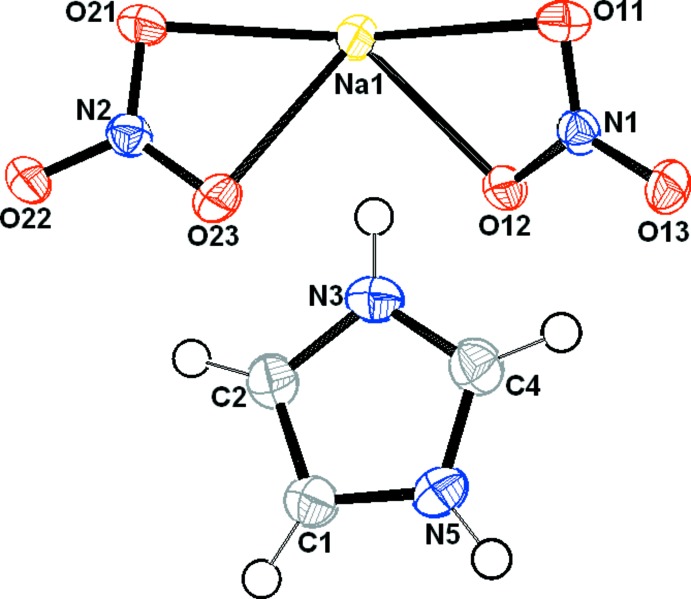
The asymetric unit of (I), with displacement ellipsoids drawn at the 50% probability level.

**Fig. 2.**
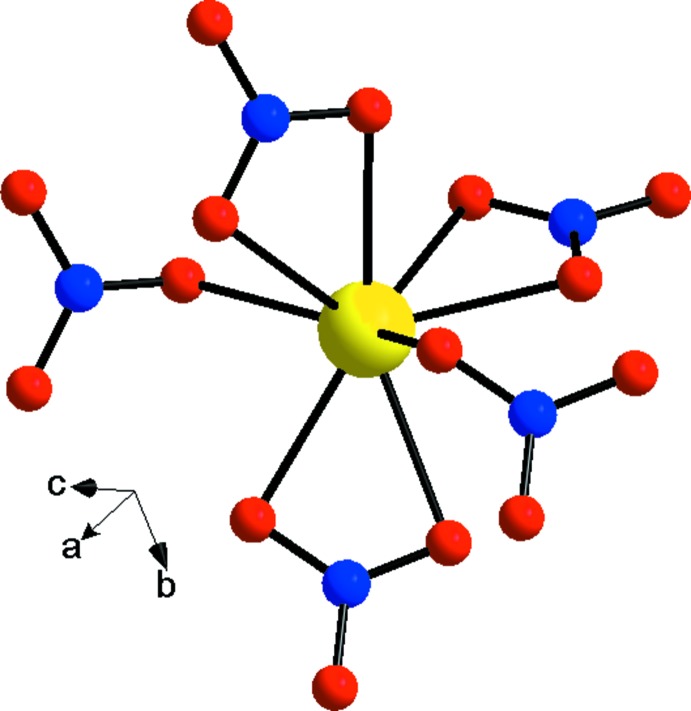
The coordination of the unieque NaI ion.

**Fig. 3.**
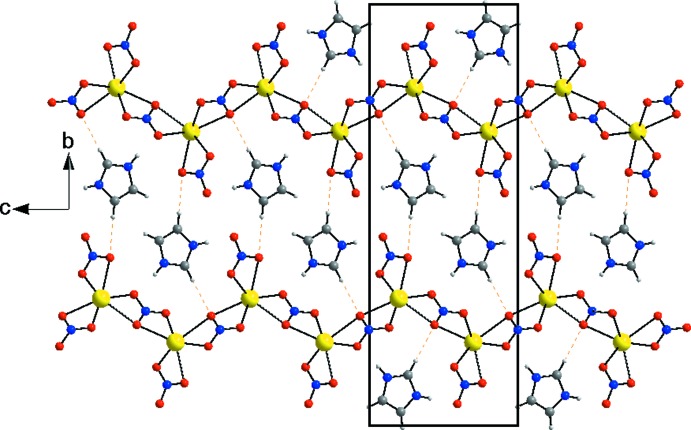
A partial packing diagram of (I), showing alterning layers with hydrogen bond shown as dashed lines.

## supplementary crystallographic information

### Comment

Knowledge of the detailed coordination behavior of imidazoles and their limitation in the possible use in complexes with specific catalytic activity is of great current importance. Imidazole, namely 1,3-diazacyclopenta-2,4-diene and its derivatives have found wide range of applications in coordination chemistry because of their multiple coordination modes as ligands to metal ions and for the construction of novel metal–organic frameworks (Huang *et al.* 2011; Huang *et al.* 2008). Recently, several complexes based on the imidazolium cation were reported (Gao *et al.* 2009; Zhang *et al.* 2011; Zhu 2012; Han *et al.* 2007; Wenyan *et al.* 2011). This paper describes the synthesis and crystal structure of the title compound.

The asymmetric unit of the title compound (I) (Fig. 1) contains one Na^I^ ion, one protonated imidazole molecule and two coordinated nitrate anions. The Na centre is coordinated by eight O atoms from three bidentate nitrate anions and two O atoms from two monodentate nitrate anions, displaying a bicapped trigonal-prismatic geometry (Fig. 2). The Na—O bond distances range from 2.4321 (16) to 2.6239 (17) Å. The C—N distances lie in the range 1.328 (2)- 1.376 (2) A°. The imidazolium cation is essentially planar giving an r.m.s. deviation for all non-H atoms of 0.0018 A°, with a maximum deviation from the mean plane of -0.0028 (1)Å for the C4 atom. The crystal packing can be described by alterning two-dimensional polymecric layers and double layers of imidazolium ions. A two-dimensional layer structure is thus constructed parallel to (010) (Fig. 3). Weak hydrogen bonds are formed between imidazolium cation and the nitrate O atoms of adjacent layers (Fig. 3) further connect the two-dimensional layers into a three-dimensional network. In addition, π···π contacts between the imidazolium rings, [centroid-centroid distance = 3.588 (3) Å with *ca* 1.382 Å slippage] are also observed.

### Experimental

All chemicals used (reagent grade) were commercially available. The compound was obtained by using hydrothermal method in Teflon-lined autoclave. The mixture of barium nitrate, imidazole, sodium hydroxide and deionized water in the molar ratio 1:1:3:264 was stirred for half an hour, and transferred in a Teflon-lined autoclave, then treated at 423 K for 4 d. After the mixture was slowly cooled to room temperature, colorless needles suitable for X-ray diffraction analysis were collected from the final reaction system by filtration, washed several times with distilled water and dried in air at ambient temperature.

### Refinement

Approximate positions for all the H atoms were first obtained from the difference electron density map. However, the H atoms were situated into idealized positions and the H-atoms have been refined with the riding-model approximation. The applied constraints were as follow: C_aryl_—H_aryl_ = 0.95 Å; N_aryl_—H_aryl_ = 0.88 Å and *U*_iso_(H_aryl_) = 1.2 *U*_eq_(C_aryl_).

### Crystal data

**Table:**

(C_3_H_5_N_2_)[Na(NO_3_)_2_]	*F*(000) = 440
*M**_r_* = 216.1	*D*_x_ = 1.834 Mg m^−^^3^
Monoclinic, *P*2_1_/*c*	Mo *K*α radiation, λ = 0.71073 Å
Hall symbol: -P 2ybc	Cell parameters from 7048 reflections
*a* = 3.5875 (3) Å	θ = 2.9–27.5°
*b* = 24.8548 (17) Å	µ = 0.22 mm^−^^1^
*c* = 8.819 (6) Å	*T* = 150 K
β = 95.546 (4)°	Stick, colourless
*V* = 782.7 (5) Å^3^	0.52 × 0.15 × 0.13 mm
*Z* = 4	

### Data collection

**Table:**

Nonius KappaCCD diffractometer	1778 independent reflections
Radiation source: Enraf Nonius FR590	1541 reflections with *I* > 2σ(*I*)
Graphite monochromator	*R*_int_ = 0.029
Detector resolution: 9 pixels mm^-1^	θ_max_ = 27.5°, θ_min_ = 4.0°
CCD rotation images, thin slices scans	*h* = −4→4
Absorption correction: multi-scan (*SADABS*; Sheldrick, 2002)	*k* = −32→32
*T*_min_ = 0.884, *T*_max_ = 0.972	*l* = −11→11
9892 measured reflections	

### Refinement

**Table:**

Refinement on *F*^2^	Primary atom site location: structure-invariant direct methods
Least-squares matrix: full	Secondary atom site location: difference Fourier map
*R*[*F*^2^ > 2σ(*F*^2^)] = 0.034	Hydrogen site location: inferred from neighbouring sites
*wR*(*F*^2^) = 0.084	H-atom parameters constrained
*S* = 1.10	*w* = 1/[σ^2^(*F*_o_^2^) + (0.0303*P*)^2^ + 0.6068*P*] where *P* = (*F*_o_^2^ + 2*F*_c_^2^)/3
1778 reflections	(Δ/σ)_max_ = 0.001
127 parameters	Δρ_max_ = 0.19 e Å^−^^3^
0 restraints	Δρ_min_ = −0.29 e Å^−^^3^

### Special details

**Table:**

Geometry. All e.s.d.'s (except the e.s.d. in the dihedral angle between two l.s. planes) are estimated using the full covariance matrix. The cell e.s.d.'s are taken into account individually in the estimation of e.s.d.'s in distances, angles and torsion angles; correlations between e.s.d.'s in cell parameters are only used when they are defined by crystal symmetry. An approximate (isotropic) treatment of cell e.s.d.'s is used for estimating e.s.d.'s involving l.s. planes.
Refinement. Refinement of *F*^2^ against ALL reflections. The weighted *R*-factor *wR* and goodness of fit *S* are based on *F*^2^, conventional *R*-factors *R* are based on *F*, with *F* set to zero for negative *F*^2^. The threshold expression of *F*^2^ > σ(*F*^2^) is used only for calculating *R*-factors(gt) *etc*. and is not relevant to the choice of reflections for refinement. *R*-factors based on *F*^2^ are statistically about twice as large as those based on *F*, and *R*- factors based on ALL data will be even larger.

### Fractional atomic coordinates and isotropic or equivalent isotropic displacement parameters (Å^2^)

**Table:**

	*x*	*y*	*z*	*U*_iso_*/*U*_eq_	
Na1	0.51191 (18)	0.19828 (2)	1.30182 (7)	0.02160 (17)	
O21	0.9994 (3)	0.13952 (4)	1.42898 (13)	0.0229 (3)	
N22	0.8825 (4)	0.09735 (5)	1.35908 (15)	0.0179 (3)	
O23	0.6569 (3)	0.10118 (5)	1.24273 (13)	0.0236 (3)	
O22	0.9921 (3)	0.05206 (4)	1.40834 (13)	0.0243 (3)	
O12	0.0537 (3)	0.18850 (4)	1.08164 (13)	0.0221 (3)	
N1	0.1514 (4)	0.22797 (5)	1.00369 (15)	0.0172 (3)	
O11	0.3871 (3)	0.26060 (4)	1.06212 (13)	0.0228 (3)	
O13	0.0153 (3)	0.23312 (5)	0.86783 (13)	0.0234 (3)	
C4	0.5075 (5)	0.12044 (6)	0.74003 (19)	0.0223 (3)	
H4	0.4512	0.1537	0.6896	0.027*	
N3	0.6807 (4)	0.11445 (5)	0.87906 (16)	0.0208 (3)	
H3	0.7619	0.1408	0.9403	0.025*	
C2	0.7128 (5)	0.06034 (6)	0.91212 (19)	0.0212 (3)	
H2	0.8252	0.0449	1.0039	0.025*	
C1	0.5545 (4)	0.03354 (6)	0.78945 (18)	0.0207 (3)	
H1	0.5348	−0.0044	0.778	0.025*	
N5	0.4268 (4)	0.07172 (5)	0.68401 (15)	0.0203 (3)	
H5	0.3101	0.065	0.5936	0.024*	

### Atomic displacement parameters (Å^2^)

**Table:**

	*U*^11^	*U*^22^	*U*^33^	*U*^12^	*U*^13^	*U*^23^
Na1	0.0229 (3)	0.0196 (3)	0.0211 (3)	0.0029 (2)	−0.0037 (3)	−0.0020 (2)
O21	0.0284 (6)	0.0159 (5)	0.0235 (6)	−0.0015 (5)	−0.0022 (5)	−0.0039 (4)
N22	0.0200 (6)	0.0169 (6)	0.0169 (7)	−0.0007 (5)	0.0019 (5)	−0.0006 (5)
O23	0.0273 (6)	0.0246 (6)	0.0178 (6)	−0.0012 (5)	−0.0046 (5)	0.0006 (4)
O22	0.0313 (7)	0.0153 (5)	0.0251 (6)	0.0018 (5)	−0.0033 (5)	0.0009 (4)
O12	0.0297 (6)	0.0169 (5)	0.0188 (6)	−0.0040 (4)	−0.0019 (5)	0.0045 (4)
N1	0.0194 (6)	0.0157 (6)	0.0165 (7)	0.0024 (5)	0.0020 (5)	−0.0005 (5)
O11	0.0225 (6)	0.0192 (5)	0.0263 (6)	−0.0052 (4)	0.0001 (5)	−0.0025 (5)
O13	0.0309 (6)	0.0239 (6)	0.0144 (6)	0.0022 (5)	−0.0024 (5)	0.0022 (4)
C4	0.0246 (8)	0.0206 (7)	0.0219 (8)	0.0004 (6)	0.0035 (6)	0.0015 (6)
N3	0.0221 (7)	0.0190 (6)	0.0214 (7)	−0.0022 (5)	0.0023 (5)	−0.0045 (5)
C2	0.0222 (8)	0.0205 (8)	0.0210 (8)	0.0012 (6)	0.0022 (6)	0.0010 (6)
C1	0.0207 (8)	0.0195 (7)	0.0219 (8)	−0.0003 (6)	0.0025 (6)	−0.0012 (6)
N5	0.0202 (7)	0.0242 (7)	0.0161 (7)	−0.0009 (5)	0.0003 (5)	−0.0025 (5)

### Geometric parameters (Å, º)

**Table:**

Na1—O12	2.4321 (16)	O12—Na1^iii^	2.8874 (17)
Na1—O21	2.4639 (14)	N1—O11	1.2467 (17)
Na1—O13^i^	2.5106 (13)	N1—O13	1.2560 (19)
Na1—O23	2.5338 (13)	N1—Na1^v^	2.9406 (16)
Na1—O13^ii^	2.5730 (14)	O11—Na1^v^	2.5910 (19)
Na1—O11^ii^	2.5910 (19)	O13—Na1^vi^	2.5106 (13)
Na1—O11	2.6239 (17)	O13—Na1^v^	2.5730 (14)
Na1—O21^iii^	2.6776 (14)	C4—N3	1.328 (2)
Na1—O12^iv^	2.8874 (17)	C4—N5	1.329 (2)
Na1—N1	2.911 (2)	C4—H4	0.95
Na1—N1^ii^	2.9406 (16)	N3—C2	1.379 (2)
Na1—Na1^iii^	3.5875 (3)	N3—H3	0.88
O21—N22	1.2668 (17)	C2—C1	1.348 (2)
O21—Na1^iv^	2.6776 (14)	C2—H2	0.95
N22—O23	1.2475 (19)	C1—N5	1.376 (2)
N22—O22	1.2558 (17)	C1—H1	0.95
O12—N1	1.2666 (17)	N5—H5	0.88
			
O12—Na1—O21	134.38 (5)	N1—Na1—N1^ii^	101.88 (5)
O12—Na1—O13^i^	131.96 (5)	O12—Na1—Na1^iii^	53.22 (4)
O21—Na1—O13^i^	80.44 (4)	O21—Na1—Na1^iii^	131.75 (3)
O12—Na1—O23	82.93 (4)	O13^i^—Na1—Na1^iii^	134.17 (3)
O21—Na1—O23	51.55 (4)	O23—Na1—Na1^iii^	103.01 (3)
O13^i^—Na1—O23	122.49 (5)	O13^ii^—Na1—Na1^iii^	44.41 (3)
O12—Na1—O13^ii^	79.49 (5)	O11^ii^—Na1—Na1^iii^	75.02 (3)
O21—Na1—O13^ii^	140.03 (5)	O11—Na1—Na1^iii^	84.68 (3)
O13^i^—Na1—O13^ii^	89.76 (4)	O21^iii^—Na1—Na1^iii^	43.36 (3)
O23—Na1—O13^ii^	146.94 (5)	O12^iv^—Na1—Na1^iii^	137.57 (3)
O12—Na1—O11^ii^	125.84 (5)	N1—Na1—Na1^iii^	69.08 (3)
O21—Na1—O11^ii^	90.37 (5)	N1^ii^—Na1—Na1^iii^	60.11 (3)
O13^i^—Na1—O11^ii^	73.07 (4)	N22—O21—Na1	94.69 (9)
O23—Na1—O11^ii^	127.91 (4)	N22—O21—Na1^iv^	117.13 (10)
O13^ii^—Na1—O11^ii^	49.87 (4)	Na1—O21—Na1^iv^	88.39 (4)
O12—Na1—O11	50.67 (4)	O23—N22—O22	120.63 (13)
O21—Na1—O11	140.80 (5)	O23—N22—O21	119.68 (13)
O13^i^—Na1—O11	81.31 (4)	O22—N22—O21	119.69 (13)
O23—Na1—O11	114.77 (5)	N22—O23—Na1	91.91 (9)
O13^ii^—Na1—O11	73.93 (5)	N1—O12—Na1	98.90 (9)
O11^ii^—Na1—O11	116.76 (4)	N1—O12—Na1^iii^	122.64 (9)
O12—Na1—O21^iii^	80.87 (5)	Na1—O12—Na1^iii^	84.35 (5)
O21—Na1—O21^iii^	88.39 (4)	O11—N1—O13	120.90 (13)
O13^i^—Na1—O21^iii^	141.34 (5)	O11—N1—O12	119.46 (13)
O23—Na1—O21^iii^	74.30 (4)	O13—N1—O12	119.62 (13)
O13^ii^—Na1—O21^iii^	75.42 (4)	O11—N1—Na1	64.35 (8)
O11^ii^—Na1—O21^iii^	70.10 (4)	O13—N1—Na1	170.21 (10)
O11—Na1—O21^iii^	126.07 (4)	O12—N1—Na1	55.64 (7)
O12—Na1—O12^iv^	84.35 (5)	O11—N1—Na1^v^	61.59 (8)
O21—Na1—O12^iv^	76.22 (5)	O13—N1—Na1^v^	60.79 (8)
O13^i^—Na1—O12^iv^	72.38 (4)	O12—N1—Na1^v^	166.49 (10)
O23—Na1—O12^iv^	67.45 (4)	Na1—N1—Na1^v^	121.45 (5)
O13^ii^—Na1—O12^iv^	137.21 (4)	N1—O11—Na1^v^	93.37 (9)
O11^ii^—Na1—O12^iv^	144.51 (4)	N1—O11—Na1	90.29 (9)
O11—Na1—O12^iv^	65.26 (5)	Na1^v^—O11—Na1	156.34 (6)
O21^iii^—Na1—O12^iv^	140.33 (4)	N1—O13—Na1^vi^	119.68 (9)
O12—Na1—N1	25.46 (4)	N1—O13—Na1^v^	93.99 (9)
O21—Na1—N1	142.68 (4)	Na1^vi^—O13—Na1^v^	89.76 (4)
O13^i^—Na1—N1	106.51 (4)	N3—C4—N5	107.85 (14)
O23—Na1—N1	97.92 (4)	N3—C4—H4	126.1
O13^ii^—Na1—N1	77.26 (5)	N5—C4—H4	126.1
O11^ii^—Na1—N1	126.91 (5)	C4—N3—C2	109.07 (14)
O11—Na1—N1	25.36 (4)	C4—N3—H3	125.5
O21^iii^—Na1—N1	104.68 (5)	C2—N3—H3	125.5
O12^iv^—Na1—N1	71.53 (5)	C1—C2—N3	106.99 (15)
O12—Na1—N1^ii^	104.05 (5)	C1—C2—H2	126.5
O21—Na1—N1^ii^	115.36 (5)	N3—C2—H2	126.5
O13^i^—Na1—N1^ii^	77.58 (4)	C2—C1—N5	106.76 (14)
O23—Na1—N1^ii^	146.03 (5)	C2—C1—H1	126.6
O13^ii^—Na1—N1^ii^	25.22 (4)	N5—C1—H1	126.6
O11^ii^—Na1—N1^ii^	25.04 (4)	C4—N5—C1	109.33 (14)
O11—Na1—N1^ii^	93.96 (5)	C4—N5—H5	125.3
O21^iii^—Na1—N1^ii^	74.13 (4)	C1—N5—H5	125.3
O12^iv^—Na1—N1^ii^	145.46 (4)		
			
O12—Na1—O21—N22	−12.65 (12)	O21—Na1—N1—O11	−102.52 (10)
O13^i^—Na1—O21—N22	−154.26 (9)	O13^i^—Na1—N1—O11	−6.60 (9)
O23—Na1—O21—N22	−8.23 (8)	O23—Na1—N1—O11	−133.96 (9)
O13^ii^—Na1—O21—N22	127.71 (9)	O13^ii^—Na1—N1—O11	79.31 (8)
O11^ii^—Na1—O21—N22	132.99 (9)	O11^ii^—Na1—N1—O11	74.35 (7)
O11—Na1—O21—N22	−90.93 (11)	O21^iii^—Na1—N1—O11	150.27 (8)
O21^iii^—Na1—O21—N22	62.90 (10)	O12^iv^—Na1—N1—O11	−71.01 (8)
O12^iv^—Na1—O21—N22	−80.21 (9)	N1^ii^—Na1—N1—O11	73.79 (10)
N1—Na1—O21—N22	−49.51 (12)	Na1^iii^—Na1—N1—O11	125.07 (8)
N1^ii^—Na1—O21—N22	134.48 (8)	O21—Na1—N1—O12	85.90 (11)
Na1^iii^—Na1—O21—N22	62.90 (10)	O13^i^—Na1—N1—O12	−178.18 (9)
O12—Na1—O21—Na1^iv^	104.45 (7)	O23—Na1—N1—O12	54.46 (9)
O13^i^—Na1—O21—Na1^iv^	−37.16 (4)	O13^ii^—Na1—N1—O12	−92.27 (9)
O23—Na1—O21—Na1^iv^	108.86 (6)	O11^ii^—Na1—N1—O12	−97.23 (10)
O13^ii^—Na1—O21—Na1^iv^	−115.19 (7)	O11—Na1—N1—O12	−171.58 (14)
O11^ii^—Na1—O21—Na1^iv^	−109.92 (4)	O21^iii^—Na1—N1—O12	−21.31 (9)
O11—Na1—O21—Na1^iv^	26.17 (8)	O12^iv^—Na1—N1—O12	117.41 (10)
O21^iii^—Na1—O21—Na1^iv^	180	N1^ii^—Na1—N1—O12	−97.78 (8)
O12^iv^—Na1—O21—Na1^iv^	36.89 (4)	Na1^iii^—Na1—N1—O12	−46.51 (8)
N1—Na1—O21—Na1^iv^	67.58 (8)	O12—Na1—N1—Na1^v^	−164.26 (12)
N1^ii^—Na1—O21—Na1^iv^	−108.43 (5)	O21—Na1—N1—Na1^v^	−78.36 (8)
Na1^iii^—Na1—O21—Na1^iv^	180	O13^i^—Na1—N1—Na1^v^	17.56 (7)
Na1—O21—N22—O23	15.20 (14)	O23—Na1—N1—Na1^v^	−109.81 (5)
Na1^iv^—O21—N22—O23	−75.40 (16)	O13^ii^—Na1—N1—Na1^v^	103.46 (6)
Na1—O21—N22—O22	−164.17 (12)	O11^ii^—Na1—N1—Na1^v^	98.51 (7)
Na1^iv^—O21—N22—O22	105.23 (14)	O11—Na1—N1—Na1^v^	24.16 (7)
O22—N22—O23—Na1	164.63 (12)	O21^iii^—Na1—N1—Na1^v^	174.43 (5)
O21—N22—O23—Na1	−14.73 (14)	O12^iv^—Na1—N1—Na1^v^	−46.85 (5)
O12—Na1—O23—N22	−174.84 (9)	N1^ii^—Na1—N1—Na1^v^	97.95 (7)
O21—Na1—O23—N22	8.34 (8)	Na1^iii^—Na1—N1—Na1^v^	149.23 (5)
O13^i^—Na1—O23—N22	49.13 (10)	O13—N1—O11—Na1^v^	−14.00 (14)
O13^ii^—Na1—O23—N22	−116.69 (11)	O12—N1—O11—Na1^v^	164.60 (12)
O11^ii^—Na1—O23—N22	−44.21 (11)	Na1—N1—O11—Na1^v^	156.62 (6)
O11—Na1—O23—N22	144.67 (9)	O13—N1—O11—Na1	−170.61 (12)
O21^iii^—Na1—O23—N22	−92.38 (10)	O12—N1—O11—Na1	7.98 (13)
O12^iv^—Na1—O23—N22	98.36 (10)	Na1^v^—N1—O11—Na1	−156.62 (6)
N1—Na1—O23—N22	164.52 (9)	O12—Na1—O11—N1	−4.67 (8)
N1^ii^—Na1—O23—N22	−70.14 (13)	O21—Na1—O11—N1	110.55 (10)
Na1^iii^—Na1—O23—N22	−125.23 (8)	O13^i^—Na1—O11—N1	173.60 (9)
O21—Na1—O12—N1	−122.22 (9)	O23—Na1—O11—N1	51.74 (9)
O13^i^—Na1—O12—N1	2.35 (12)	O13^ii^—Na1—O11—N1	−94.12 (9)
O23—Na1—O12—N1	−125.70 (9)	O11^ii^—Na1—O11—N1	−120.42 (7)
O13^ii^—Na1—O12—N1	82.42 (9)	O21^iii^—Na1—O11—N1	−36.40 (10)
O11^ii^—Na1—O12—N1	101.91 (10)	O12^iv^—Na1—O11—N1	99.07 (9)
O11—Na1—O12—N1	4.65 (8)	N1^ii^—Na1—O11—N1	−109.62 (10)
O21^iii^—Na1—O12—N1	159.15 (9)	Na1^iii^—Na1—O11—N1	−50.16 (8)
O12^iv^—Na1—O12—N1	−57.79 (9)	O12—Na1—O11—Na1^v^	−103.77 (14)
N1^ii^—Na1—O12—N1	88.16 (7)	O21—Na1—O11—Na1^v^	11.44 (17)
Na1^iii^—Na1—O12—N1	122.21 (9)	O13^i^—Na1—O11—Na1^v^	74.50 (12)
O21—Na1—O12—Na1^iii^	115.57 (6)	O23—Na1—O11—Na1^v^	−47.37 (14)
O13^i^—Na1—O12—Na1^iii^	−119.86 (5)	O13^ii^—Na1—O11—Na1^v^	166.77 (13)
O23—Na1—O12—Na1^iii^	112.09 (4)	O11^ii^—Na1—O11—Na1^v^	140.47 (11)
O13^ii^—Na1—O12—Na1^iii^	−39.79 (4)	O21^iii^—Na1—O11—Na1^v^	−135.51 (12)
O11^ii^—Na1—O12—Na1^iii^	−20.30 (6)	O12^iv^—Na1—O11—Na1^v^	−0.03 (12)
O11—Na1—O12—Na1^iii^	−117.56 (5)	N1—Na1—O11—Na1^v^	−99.10 (15)
O21^iii^—Na1—O12—Na1^iii^	36.93 (4)	N1^ii^—Na1—O11—Na1^v^	151.28 (12)
O12^iv^—Na1—O12—Na1^iii^	180	Na1^iii^—Na1—O11—Na1^v^	−149.26 (12)
N1—Na1—O12—Na1^iii^	−122.21 (9)	O11—N1—O13—Na1^vi^	−77.90 (15)
N1^ii^—Na1—O12—Na1^iii^	−34.05 (4)	O12—N1—O13—Na1^vi^	103.51 (13)
Na1—O12—N1—O11	−8.72 (14)	Na1^v^—N1—O13—Na1^vi^	−92.00 (8)
Na1^iii^—O12—N1—O11	80.25 (15)	O11—N1—O13—Na1^v^	14.11 (14)
Na1—O12—N1—O13	169.89 (11)	O12—N1—O13—Na1^v^	−164.48 (12)
Na1^iii^—O12—N1—O13	−101.14 (14)	N5—C4—N3—C2	0.31 (19)
Na1^iii^—O12—N1—Na1	88.97 (9)	C4—N3—C2—C1	0.00 (19)
Na1—O12—N1—Na1^v^	82.0 (4)	N3—C2—C1—N5	−0.30 (18)
Na1^iii^—O12—N1—Na1^v^	170.9 (4)	N3—C4—N5—C1	−0.50 (19)
O12—Na1—N1—O11	171.58 (14)	C2—C1—N5—C4	0.50 (18)

Symmetry codes: (i) *x*+1, −*y*+1/2, *z*+1/2; (ii) *x*, −*y*+1/2, *z*+1/2; (iii) *x*−1, *y*, *z*; (iv) *x*+1, *y*, *z*; (v) *x*, −*y*+1/2, *z*−1/2; (vi) *x*−1, −*y*+1/2, *z*−1/2.

### Hydrogen-bond geometry (Å, º)

**Table:**

*D*—H···*A*	*D*—H	H···*A*	*D*···*A*	*D*—H···*A*
C1—H1···O23^vii^	0.95	2.50	3.438 (3)	168
C4—H4···O11^v^	0.95	2.41	3.355 (3)	173

Symmetry codes: (v) *x*, −*y*+1/2, *z*−1/2; (vii) −*x*+1, −*y*, −*z*+2.
